# High Prevalence of Metabolic Dysfunction-Associated Steatotic Liver Disease in Patients with Hidradenitis Suppurativa: A Guide to Easily Assess the Clinical Risk of Comorbid Liver Disease

**DOI:** 10.1016/j.xjidi.2025.100419

**Published:** 2025-09-22

**Authors:** Verena G. Frings, Maxine Gläsel, Monika Rau, Andreas Geier, Janik Fleißner, Dagmar Presser, Matthias Goebeler, Andreas Kerstan

**Affiliations:** 1 Department of Dermatology, Venereology and Allergology, University Hospital Würzburg, Würzburg, Germany; 2 Department of Medicine II, Hepatology, University Hospital Würzburg, Würzburg, Germany

**Keywords:** Acne inversa, Liver steatosis, MASLD, Nonalcoholic fatty liver disease, Transient elastrography

## Abstract

Hidradenitis suppurativa (HS) is a chronic inflammatory skin condition associated with considerable comorbidity. The link between HS and metabolic dysfunction–associated steatotic liver disease (MASLD) is of particular interest owing to shared inflammatory pathways. This study applies the new MASLD nomenclature in a HS cohort. Our study aims to investigate the prevalence of MASLD in HS using transient elastography and to develop a clinical algorithm for assessing the MASLD risk. A cross-sectional study was conducted involving 94 patients with HS. Noninvasive methods were employed to diagnose MASLD. The clinical diagnosis was based on altered transient elastography–controlled attenuation parameter as surrogate for liver steatosis and the presence of cardiometabolic risk factors after excluding secondary causes of steatosis. Statistical analyses included logistic regression models to identify predictors of MASLD risk. The study found a prevalence of MASLD as high as 75% measured by high-accuracy transient elastography among patients with HS. Multivariable logistic regression analysis showed a strong within-cohort association between HS and MASLD. The newly developed clinical algorithm integrating transient elastography and the Fatty Liver Index effectively stratified MASLD risk. Our findings underscore the importance of routine MASLD screening in HS. The proposed clinical algorithm offers a straightforward approach for assessing MASLD risk in HS.

## Introduction

Metabolic dysfunction–associated steatotic liver disease (MASLD), previously known as nonalcoholic fatty liver disease, is the most common chronic liver disease worldwide. MASLD may affect any age group and has been described in most ethnic backgrounds (Blond et al, 2017). With an estimated prevalence of about 30% in Western countries mainly due to sedentary lifestyles and high-fat diets (Younossi, 2019; Younossi and Henry, 2021), MASLD constitutes a serious public health concern. MASLD is defined as the presence of hepatic steatosis in conjunction with at least 1 cardiometabolic risk factor (CMRF) in the absence of increased alcohol intake (Rinella et al, 2023). The metabolic syndrome characterized by obesity, type 2 diabetes mellitus (T2DM), arterial hypertension, and hyperlipidemia appears to play a pivotal role for the development of MASLD (Kneeman et al, 2012). Especially hypertriglyceridemia (rather than hypercholesterolemia) may increase the risk of MASLD (Angulo, 2002; Angulo and Lindor, 2002). Individuals with MASLD and steatohepatitis are designated as having metabolic dysfunction–associated steatohepatitis. MASLD comprises a wide spectrum of liver damage and may result in end-stage liver disease, including hepatocellular carcinoma (Rinella et al, 2023).

There is accumulating evidence that hidradenitis suppurativa (HS) (also known as acne inversa) (Dréno et a., 2012; Wolk et al, 2020; Zouboulis et al, 2015) is a systemic inflammatory condition extending beyond the skin with a wide range of comorbidities, including cardiovascular, endocrinological, and psychological disorders (Nguyen et al, 2021; Sabat et al, 2020; Tzellos and Zouboulis, 2022). Although the mechanisms underlying HS are complex and involve innate and adaptive immunity imbalances (Frings et al, 2022; Lima et al, 2016; Sabat et al, 2020), HS is characterized by the increased secretion of proinflammatory cytokines, such as IL-17 and TNFα. The contribution of IL-17 to the pathogenesis of both HS and MASLD is intriguing. IL-17A–secreting T helper 17 cells may promote the progression from simple steatosis to steatohepatitis (Tang et al, 2011). Likewise, T helper 17 cells can be detected in adipose tissue, and IL-17 itself regulates glucose metabolism and adipogenesis. However, to date, high-level evidence addressing the independent association between MASLD and HS is sparse (Bailey et al, 2022; Durán-Vian et al, 2019; Gau et al, 2022; Omari et al, 2025). Comorbidity screening in HS indicated an unsure association between HS and MASLD, and therefore, no screening has been recommended yet (Garg et al, 2022).

Determining liver blood parameters and performing transient elastography (TE) to assess liver steatosis and fibrosis, we observed a strong association between HS and MASLD. Our data highlight the importance of screening for MASLD in patients with HS and permitted the development of an algorithm to easily assess their MASLD risk. To the best of our knowledge, this study applied the new MASLD nomenclature in the investigation of an HS cohort.

## Results

### Elevated risk of MASLD in patients with HS

**Figure 1:**
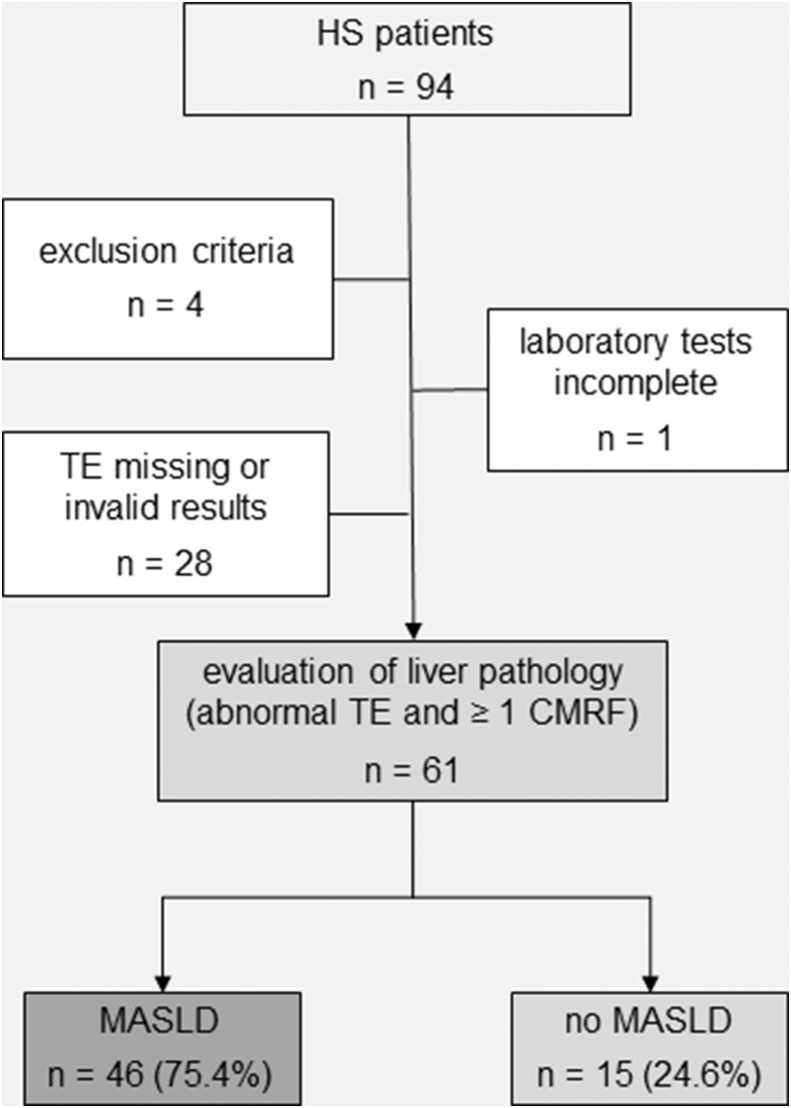
**Participants.** Among the initially 94 screened patients with HS, a total of 61 patients were finally included in the study. Anthropometric, clinical, and laboratory variables were collected. Clinical diagnosis of MASLD was established on the basis of the presence of abnormal TE in conjunction with at least 1 CMRF, once secondary causes of steatosis were ruled out. HS, hidradenitis suppurativa; CMRF, cardiometabolic risk factor; MASLD, metabolic dysfunction–associated steatotic liver disease; TE, transient elastography.

This study screened 94 patients with HS who visited the outpatient clinic of the Department of Dermatology, University Hospital Würzburg between September 2020 and July 2022. Baseline characteristics of 61 included patients with HS are shown in Table 1. On the basis of the TE measurements that allowed to noninvasively measure liver stiffness and the presence of at least 1 CMRF, the group was divided into HS with MASLD (HS + MASLD) and HS without MASLD (HS − MASLD) as depicted in Figure 1. There were no cases of cryptogenic liver disease (steatosis in the absence of any CMRF) in the studied cohort. The group HS + MASLD was on average older and had a significantly higher body mass index (BMI) (*P* < .001). Similarly, for both males and females, the waist circumferences were significantly higher in the HS + MASLD cohort. No significant differences were found between the HS ± MASLD subgroups with regard to age at disease onset, days absent from work, life quality, or disease severity.

**Table 1:** Sociodemographic and Clinical Characteristics of the HS Cohort

Characteristics	All HS	HS + MASLD	HS − MASLD	*P*-Value
n (%)	61 (100)	46 (75)	15 (24.6)	
Sex, n (%)				
Men	26 (43)	20 (44)	6 (40)	
Women	35 (57)	26 (57)	9 (60)	
Age, y, mean ± SD	37.9 ± 11.1	39.6 0 ± 10.0	32.6 ± 12.9	.069
BMI, kg/m^2^, mean ± SD	30.0 ± 5.8	31.5 ± 5.2	25.5 ± 5.4	<.001
Normal weight, n (%)	13 (21)	5 (11)	8 (53)	
Overweight, n (%)	18 (30)	13 (28)	5 (33)	
Obesity grade 1, n (%)	18 (30)	17 (37)	1 (7)	
Obesity grade 2, n (%)	8 (13)	8 (17)	0	
Obesity grade 3, n (%)	4 (7)	3 (7)	1 (7)	
Waist circumference, cm, mean ± SD^1^				
Men	104.3 ± 13.6	108.8 ± 12.1	89.5 ± 5.6	<.001
Women	93.6 ± 13.4	97.5 ± 11.5	82.3 ± 12.8	.002
Age at disease onset, y, mean ± SD	23.9 ± 10.4	25.1 ± 10.9	20.2 ± 8.1	.05
Absence from work, d, mean ± SD	109.7 ± 186.8	133.9 ± 208.6	36.8 ± 51.6	.098
DLQI, mean ± SD	9.7 ± 7.9	10.5 ± 7.8	7.1 ± 7.9	.168
Hurley stage, n (%)				.400
Stage I	13 (21)	9 (20)	4 (27)	
Stage II	31 (51)	23 (50)	8 (53)	
Stage III	17 (28)	14 (30)	3 (20)	
IHS4, mean ± SD	5.5 ± 6.3	6.0 ± 6.7	4.1 ± 4.5	.231
MSS, mean ± SD	17.5 ± 19.7	19.4 ± 21.5	11.9 ± 11.5	.091

Abbreviations: BMI, body mass index; DLQI, Dermatology Life Quality Index; HS, hidradenitis suppurativa; IHS4, International Hidradenitis Suppurativa Severity Scoring System; MASLD, metabolic dysfunction–associated steatotic liver disease; MSS, modified Sartorius Score.

Statistical significance was calculated between HS + MASLD and HS – MASLD. *t*-test was used for equality of means, and Levene test was used for equality of variances.

1 Waist circumferences standard values: males: ≤102 cm; females: ≤88 cm.

In the HS + MASLD cohort, a significant difference for arterial hypertension (*P* = .006) and dyslipidemia characterized by abnormally high or low amounts of any or all lipids (*P* = .007) was registered (Table 2). Ninety-five percent of the patients with HS with dyslipidemia had MASLD (*P* = .007). All of the patients in the HS + MASLD group fulfilled the criteria for metabolic syndrome (*P* = .029). A significant difference was found in γ-glutamyltransferase (γ-GT) and total cholesterol fasting blood levels (*P* < .01 and *P* = .02, respectively). γ-GT values of the HS + MASLD group were often in the high-normal range, and cholesterol levels were higher in the HS + MASLD group than in the HS – MASLD group. Platelet counts and albumin levels, parameters impaired in case of compromised liver function, were within the normal range.

**Table 2:** Pre-Existing Conditions and Laboratory Values of the HS Cohort

	All HS	HS + MASLD	HS − MASLD	*P*-Value
n (%)	61 (100)	46 (75)	15 (25)	
Pre-existing conditions, n (%)				
Arterial hypertension	17 (28)	17 (100)	0	.006
T2DM	7 (12)	7 (100)	0	.111
Dyslipidemia^1^	22 (36)	21 (96)	1 (5)	.007
Hypothyreosis	12 (20)	9 (75)	3 (25)	.971
Metabolic syndrome^2^, n (%)	12 (20)	12 (100)	0	.029
Laboratory values (mean ± SD)				
AST, U/l	23.9 ± 10.4	24.3 ± 10.6	22.5 ± 9.6	.539
ALT, U/l	27.5 ± 23.9	29.5 ± 25.7	21.4 ± 15.9	.169
γ-GT, U/l	25.4 ±17.8	28.5 ± 18.9	15.8 ± 7.8	<.01
Cholesterol total, mg/dl	186.2 ± 43.3	193.3 ± 45.6	164.3 ± 24.6	.02
HDL-cholesterol, mg/dl	51.0 ± 13.0	50.0 ± 13.0	56.4 ± 12.6	.099
LDL-cholesterol, mg/dl	104.0 ± 37.2	109.0 ± 40.0	88.3 ± 20.4	.012
Triglycerides, mg/dl	156.0 ± 85.6	175.0 ± 88.4	97.9 ± 36.2	.002
Platelets, 10^−9^/l	292.4 ± 67.3	294.9 ± 56.7	284.7 ± 92.1	.698
Albumin, g/dl	4.5 ± 0.3	4.5 ± 0.3	4.6 ± 0.2	.456
HbA1c^c^, %	5.8 ± 0.8	5.9 ± 0.9	5.5 ± 0.2	.194
Fasting glucose^3^, mg/dl	95.4 ± 14.9	96.0 ± 16.0	92.5 ± 8.8	.799

Abbreviations: γ-GT, γ-glutamyltransferase; ALT, alanine aminotransferase; AST, aspartate aminotransferase; ATP III, Adult Treatment Panel III; HDL, high-density lipoprotein; HS, Hidradenitis suppurativa; MASLD, metabolic dysfunction–associated steatotic liver disease; T2DM, type 2 diabetes mellitus.

Statistical significance was calculated between HS + MASLD and HS − MASLD; for laboratory values, the *t*-test for equality of means and the Levene test for equality of variance were used; for pre-existing conditions, the Man–Whitney *U* test was employed. Limit values used: AST (U/l): male 10–50, female ≤35; ALT (U/l): male 10–50, female ≤35. γ-GT (U/l): male ≤ 60, female ≤ 40; cholesterol (mg/dl): 130–220; HDL cholesterol (mg/dl): ≥35; LDL cholesterol (mg/dl): 0–150; triglycerides (mg/dl): 74–172; platelets (10^−9^/l): male 151–304, female 150–450; albumin (g/dl): male 3.5–5.2, female 3.5–5.5; HbA1c (%): <6.5; and fasting glucose (mg/dl): 74–106.

1 According to in-house measured laboratory values.

2 Metabolic syndrome according to the ATP II criteria (Lipsy, 2003): abdominal obesity, high triglycerides, low HDL cholesterol, high blood pressure, high fasting glucose.

3 In n = 2 patients, neither an HbA1c value nor a fasting glucose value was available.

The average controlled attenuation parameter (CAP) value, measured by TE, was 305 db/m (±43.9) in the HS + MASLD cohort and 201 db/m (±22.3) in the HS − MASLD cohort (*P* < .001) (Table 3). Grade 3 steatosis was most common with 63% (29 patients), in contrast to grade 1 in 13 patients (28.3%) and grade 2 in 4 patients (8.7%). On average, liver stiffness was in the physiological range in both cohorts and did not differ significantly. No case of fibrosis was recorded in the HS − MASLD cohort. In contrast, TE registered grades 2–3 fibrosis in 13% (6 patients) with HS + MASLD and advanced grade 4 fibrosis in 1 case. Overall, the prevalence of fibrosis > grade 2 was 15% in the HS + MASLD cohort (Table 3).

**Table 3:** Liver TE and Noninvasive Fibrosis Scores

	HS + MASLD	HS − MASLD	*P*-Value
CAP, db/m, mean ± SD	305.4 ± 43.9	201.4 ± 22.3	<.001
Steatosis, n (%)			
Grade 1	13 (28)	—	
Grade 2	4 (9)	—	
Grade 3	29 (63)	—	
Liver stiffness, kPa, mean ± SD	5.5 ± 2.4	4.7 ± 0.6	.441
Fibrosis, n (%)			
Grade ≤1	39 (85)	—	
Grades 2–3	6 (13)	—	
Grade 4	1 (2)	—	
FLI, mean ± SD	67.7 ± 28.1	24.0 ± 23.4	<.001
NAFLD fibrosis score, mean ± SD	−2.9 ± 1.3	3.6 ± 1.5	.088
Fib-4, mean ± SD	0.7 ± 0.2	0.7 ± 0.6	.269

Abbreviations: CAP, controlled attenuation parameter; FLI, Fatty Liver Index; HS, hidradenitis suppurativa; MASLD, metabolic dysfunction–associated steatotic liver disease; NAFLD, nonalcoholic fatty liver disease; TE, transient elastography.

For CAP values and the NAFLD Fibrosis Score, the *t*-test for equality of means and the Levene test for equality of variance were used; regarding Fib-4, FLI, and liver stiffness, the Man–Whitney *U* test was employed.

### Validation of clinical scores for the evaluation of liver pathologies in HS

Different clinical scores for risk assessment of liver pathologies in clinical routine were investigated regarding their usability and accuracy. Neither the nonalcoholic fatty liver disease fibrosis score (NFS) nor the Fib-4 index showed satisfactory sensitivity values (Table 4). The Fatty Liver Index (FLI) was the only score to show statistically significant divergences between the HS ± MASLD cohorts, not exceeding physiological range <30 points in the HS − MASLD cohort (Table 3). Sensitivity and specificity were determined according to the cutoff values listed in Table 4. A cutoff of 20 points was identified as the most suitable for the detection of MASLD in patients with HS for the FLI.

**Table 4:** Cardiometabolic Risk Factors and MASLD Diagnostic Criteria

BMI ≥24 kg/m^2^ or WC >90 cm (male)/>80 cm (female)
Fasting serum glucose ≥5.6 mmol/l (100 mg/dl) or 2-hour postload glucose levels ≥7.8 mmol/l (140 mg/dl) or HbA1c ≥5.7% (39 mmol/l) or T2DM or treatment for T2DM
Blood pressure ≥130/85 mmHg or specific antihypertensive drug treatment
Plasma triglycerides ≥1.70 mmol/l (150 mg/dl) or lipid lowering treatment
Plasma HDL-cholesterol ≤1.0 mmol/l (40 mg/dl) (male)/≤1.3 mmol/l (50 mg/dl) (female) or lipid lowering treatment

Abbreviations: BMI, body mass index; HDL, high-density lipoprotein; MASLD, metabolic dysfunction–associated steatotic liver disease; T2DM, type 2 diabetes mellitus; WC, waist circumference.

In the presence of hepatic steatosis, the finding of any cardiometabolic risk factor would indicate a diagnosis of MASLD if there are no other causes of hepatic steatosis (Hong et al, 2024).

**Figure 2:**
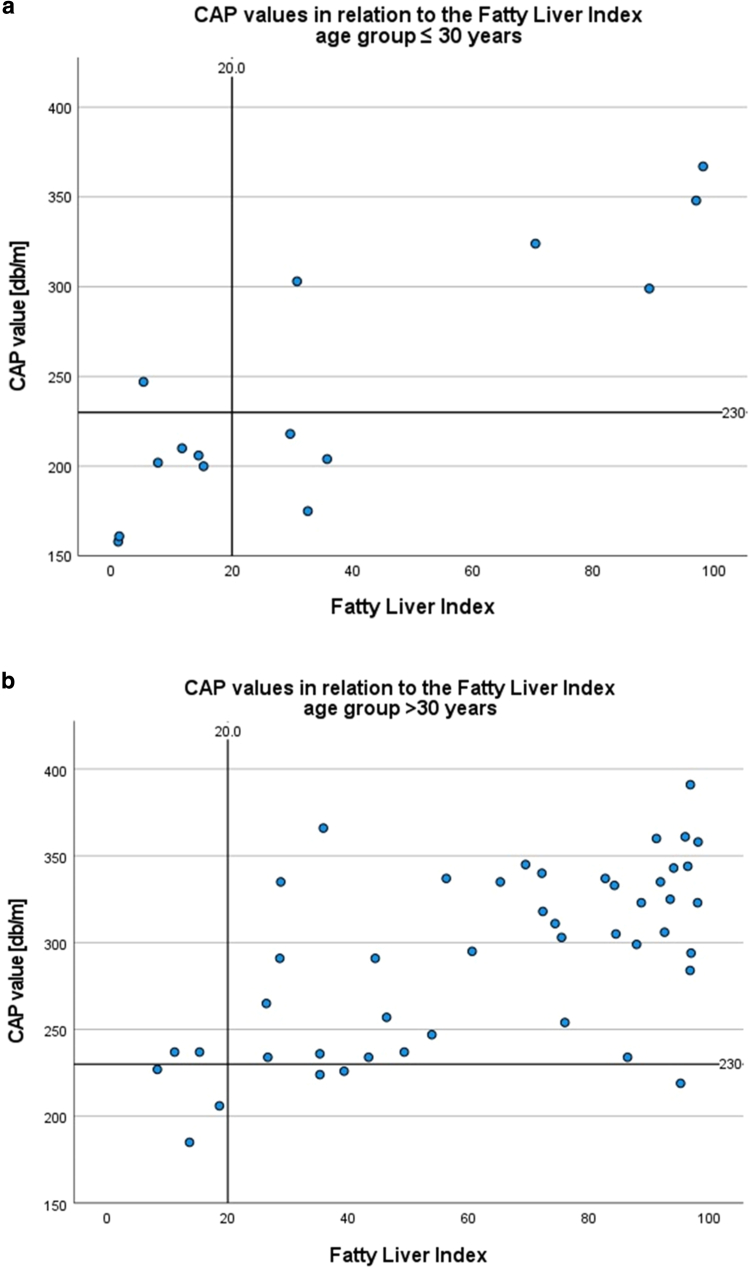
**Scatter plot showing the results of the FLI in relation to the level of steatosis.** Results (indicated by CAP value) are for the age groups (**a**) ≤30 years and (**b**) >30 years with a cutoff of 20 points and, therefore, the highest measured sensitivity. CAP, controlled attenuation parameter; FLI, Fatty Liver Index.

In addition, we aimed to check for an age-dependent calculation of the FLI test quality criteria and whether a higher cutoff value should be used for patients aged ≤30 years. For the age group >30 years, the best sensitivity with 95% and specificity with 50% could be calculated for a cutoff at 20 points, with a positive predictive value of 0.93. For the age group ≤30 years, a cutoff of 40 points had the highest positive predictive value of 1.0 (Figure 2). However, without age adjustment, a cutoff of 20 points had the highest sensitivity of all tested cutoff values at 88% (compare Figure 2a), which seemed to be most accurate for the total cohort.

### Evaluation of HS as risk factor for developing MASLD

**Figure 3:**
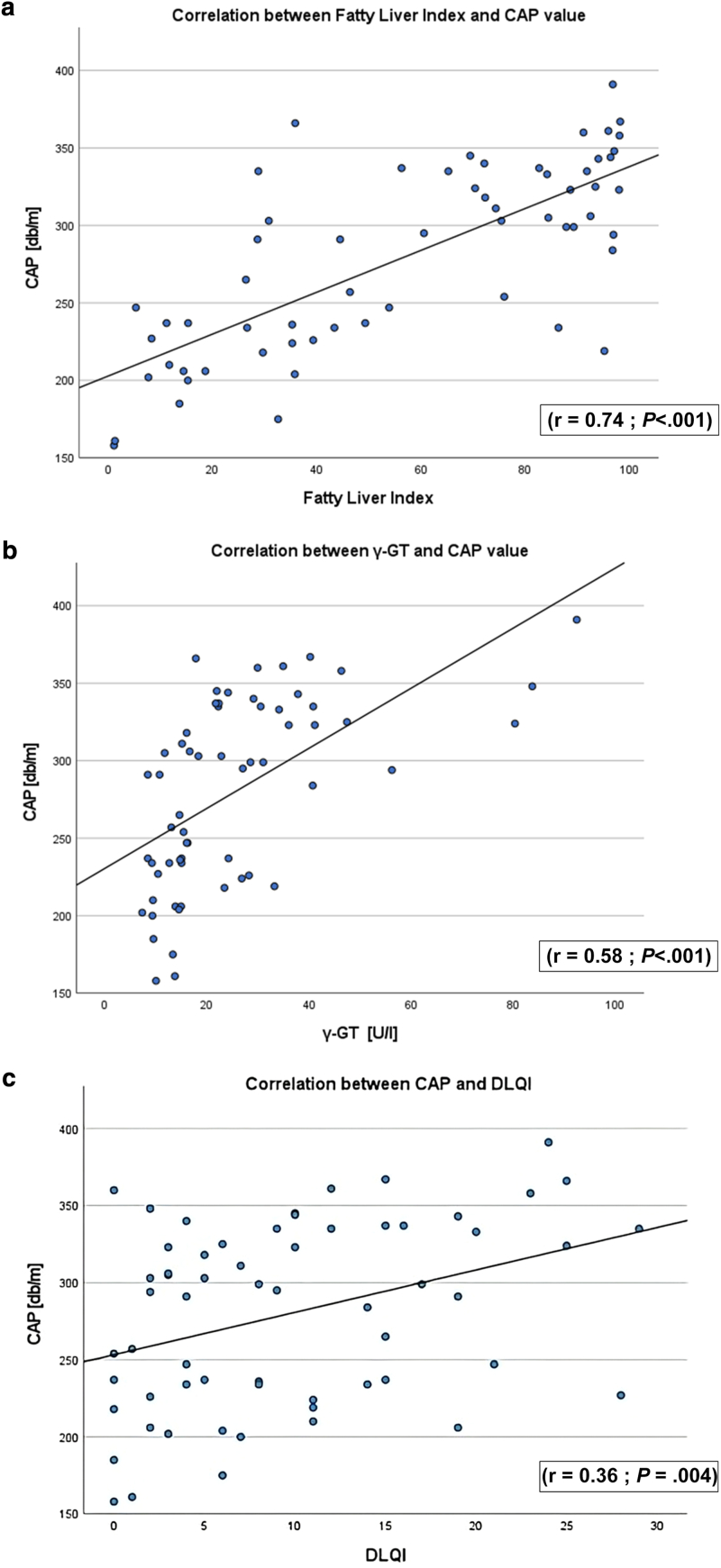
**Correlation between CAP value and FLI, γ-GT, and DLQI.** Scatter plots showing the correlation between (**a**) FLI, (**b**) γ-GT, and (**c**) DLQI with the level of steatosis as reflected by the CAP value. Pearson correlation and *t*-test were used. γ-GT, γ-glutamyltransferase; CAP, controlled attenuation parameter; DLQI, Dermatology Life Quality Index; FLI, Fatty Liver Index.

**Figure 4:**
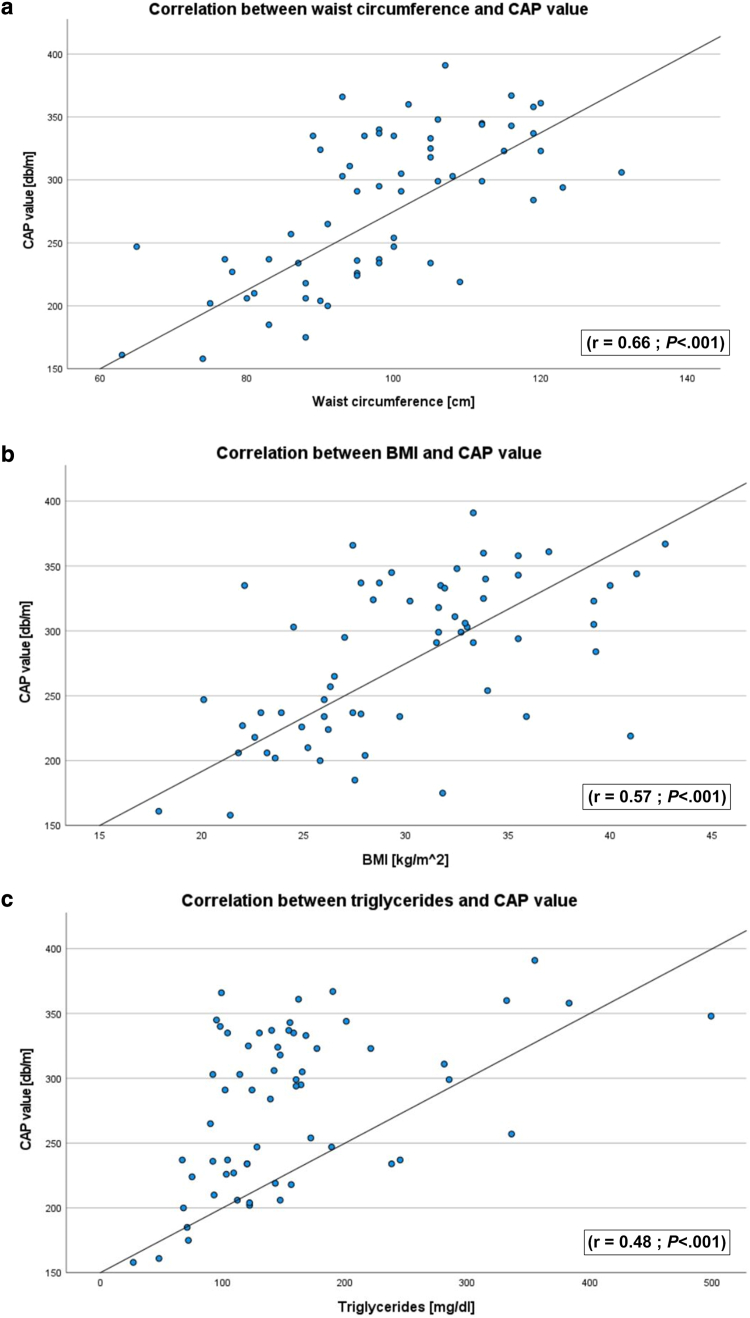
**Correlation between CAP value and triglycerides, waist circumference, and BMI.** Shown are scatter plots showing the correlation between (**a**) triglycerides, (**b**) waist circumference, and (**c**) BMI with the level of steatosis as indicated by the CAP value. Pearson correlation and *t*-test were used. BMI, body mass index; CAP, controlled attenuation parameter.

Within the HS cohort, the CAP value correlated positively and significantly with the FLI (r = 0.71; *P* < .001; 95% confidence interval [CI] = 0.55–0.82) (Figure 3a), Dermatology Life Quality Index (r = 0.36; *P* = .004; 95% CI = 0.12–0.56) (Figure 3c), waist circumference (r = 0.66; *P* < .001; 95% CI = 0.49–0.78) (Figure 4a), and BMI (r = 0.57; *P* < .001; 95% CI = 0.38–0.72) (Figure 4b). Regarding laboratory values, total cholesterol (r = 0.32; *P* = .011; 95% CI = 0.08–0.53) (data not shown) and triglycerides (r = 0.48; *P* < .001; 95% CI = 0.26–0.66) (Figure 4c) showed a correlation with a medium effect and significant *P*-value. The strongest effect regarding laboratory values is shown by γ-GT (r = 0.58; *P* < .001; 95% CI = 0.38–0.72) (Figure 3b). However, all other variables that were required to calculate the FLI (triglycerides, BMI, waist circumference) also correlated positively and significantly with the CAP value (Figure 4a–c).

Multivariable logistic regression analysis investigated the influence of HS on the extent of fatty liver disease (Table 5). It was shown that the variable "metabolic risk factor" had the highest influence on the severity of MASLD of all the variables included, highlighting the adaption of the new nomenclature. In the age cohort <45 years, the presence of 1 or more metabolic risk factors changed the CAP value by as much as 61.5 dB/m. In the younger age group, the presence of HS as well as male sex also had an influence on CAP values.

**Table 5:** Test Quality Criteria of the NAFLD Fibrosis Score for Different Cutoff Values

	Cutoff Value	Sensitivity	Specificity	PPV	NPV	Accuracy
NAFLD Fibrosis Score	−1.455	14%	93%	0.2	0.89	0.84
	0.676	14%	100%	1.0	0.9	0.90
Fib-4 (Shah et al, 2011)	1.45	0%	98%	0	0.88	0.87
	2.67	0%	98%	0	0.88	0.87
Fatty Liver Index (Bedogni et al, 2006)	20	94%	60%	0.88	0.75	0.85
	30^1^	85%	67%	0.89	0.59	0.80
	40	78%	93%	0.97	0.58	0.82
	60	65%	93%	0.97	0.47	0.72

Abbreviations: NAFLD, nonalcoholic fatty liver disease; NPV, negative predictive value; PPV, positive predictive value.

1 In the original literature (Bedogni et al, 2006), a cutoff of 30 points is used to rule out fatty liver. The frequency distribution was calculated using a contingency table.

### MASLD screening algorithm for patients with HS

**Figure 5:**
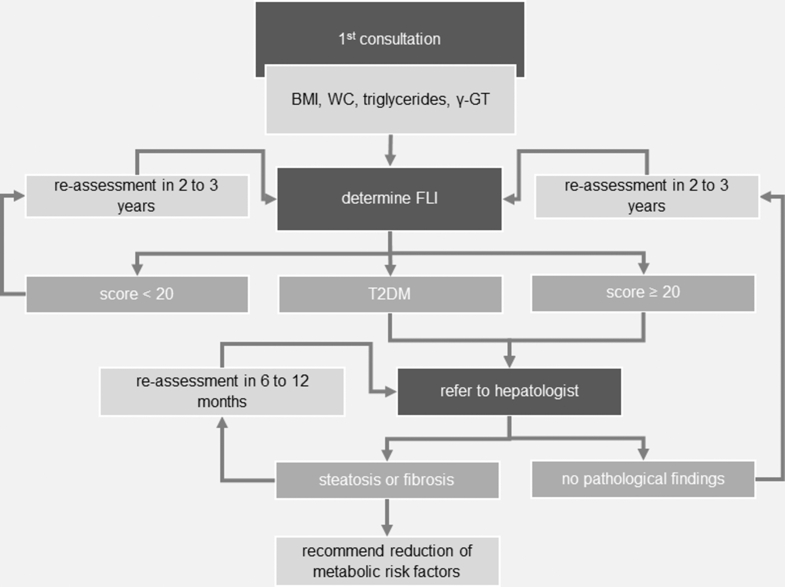
**MASLD screening algorithm for patients with HS.** γ-GT, γ-glutamyltransferase; BMI, body mass index; FLI, Fatty Liver Index; HS, hidradenitis suppurativa; MASLD, metabolic dysfunction*–*associated steatotic liver disease; T2DM, type 2 diabetes mellitus; WC, waist circumference.

On the basis of the results presented, we created a decision tree for MASLD screening in HS (Figure 5). At first consultation, BMI, waist circumference, fasting triglycerides, and γ-GT should be determined. Then, FLI calculation is recommended. Patients with <20 points have a low risk of liver pathology. On the basis of the current European guideline on MASLD (European Association for the Study of the Liver, 2024) but not directly evaluated or validated in our study population, risk assessments may be repeated every 2–3 years. Patients scoring ≥20 are encouraged to consult an hepatologist. If T2DM is present, referral to a hepatologist should be recommended immediately because 100% of patients with T2DM with HS suffered from MASLD in our cohort. If hepatological findings are unremarkable, a new risk stratification can be carried out every 2–3 years. If hepatological consultation shows steatosis or fibrosis, patients are generally advised to reduce metabolic risk factors, and further treatment options should be discussed.

## Discussion

In the cohort of patients with HS presented in this study, the prevalence of MASLD was 75% as measured by high-accuracy TE and evaluation of CMRFs. This number is more than twice as high as the estimates for the overall population in Western industrialized countries (Masarone et al, 2014; Younossi et al, 2016). In a recent meta-analysis, the prevalence of MASLD in HS cohorts varied between 38.5 and 73%. All studies supported the hypothesis that HS favors MASLD development (Bailey et al, 2022; Omari et al, 2025; Powell et al, 2021). In terms of sensitivity, TE is superior to abdominal ultrasonography (Roeb et al, 2022; Williams et al, 2011). This may explain why, apart from us, the only other study using TE observed a comparably high MASLD prevalence of 73% (Durán-Vian et al, 2019). Within the HS + MASLD cohort, advanced fibrosis was diagnosed in about 15% of the participants, exceeding the estimated overall prevalence of 4% in the normal population (Estes et al, 2018) and opposing current literature on HS cohorts (Omari et al, 2025). One study suspected a correlation between the severity of HS and the severity of fatty liver disease (Durán-Vian et al, 2019), which is not supported by our data that included the Hurley score and modified Sartorius Score. Interestingly, no cases of cryptogenic liver disease (altered CAP but no CMRFs) were noted in our cohort, further supporting the link between HS, CMRFs, and liver disease.

Our study identified clinical predictors associated with MASLD within the HS cohort, and therefore, HS proved to be in strong association with MASLD, as also observed in other studies (Durán-Vian et al, 2019; González-Villanueva et al, 2020). In line with Durán-Vian et al (2019), who identified male sex as a protective factor, this study showed that male sex has a favorable effect on MASLD. In the presence of just 1 metabolic risk factor, the CAP value increased by 52–62 dB/m, which could result in a worse degree of steatosis.

The association between HS and liver pathology is hypothesized to result from chronic systemic inflammation in HS due to increased levels of adipokines (Malara et al, 2018), which regulate hepatic lipid accumulation and entail greater insulin resistance. In addition, proinflammatory cytokines (IL-1, IL-6, IL-23, TNF) and the imbalance in T-cell subtypes, such as T helper 17/T regulatory cell, promote hepatic inflammation and induction of fibrosis, leading to the potential development and progression of liver disease (Powell et al, 2021; Crespo et al, 2001). This is also supported by the findings that 61% of patients with HS + MASLD were obese, and BMI was significantly higher in the HS + MASLD cohort than in the HS − MASLD group (*P* < .001). Obesity is a pathogenetic driver of MASLD (Roeb et al, 2022), and our study data thus reflect this notion. Given the relatively young age at which patients with HS typically develop the disease, they are at an increased risk of MASLD over an extended time period, owing to both the HS itself and associated metabolic disorders (Roeb et al, 2022). Patients with MASLD have also been shown to have an increased risk of developing T2DM owing to the heightened insulin resistance associated with fatty liver (Duan et al, 2022). In this study, only 15% of patients with HS + MASLD suffered from T2DM, whereas 100% of patients with HS + T2DM had MASLD. The comparatively low prevalence of T2DM in the HS + MASLD cohort may be explained by the young age of the patients. The average age of onset for patients with T2DM is between 45 and 65 years, with a prevalence rate of 4.7% (Kovács et al, 2024). Given these figures, the prevalence of T2DM among patients with HS is above average. Fasting glucose showed a moderately significant correlation with CAP, reflecting the increased insulin resistance characteristic of MASLD (Powell et al, 2021). This underscores the necessity to specifically screen for diabetes mellitus on a routine basis and to initiate TE if diagnosis is confirmed (Roeb et al, 2022).

Interestingly, the Dermatology Life Quality Index in the HS study population exhibited a medium correlation with the CAP value (r = 0.36; *P* = .004) (data not shown). This is particularly noteworthy because steatosis itself does not cause any specific symptoms (Roeb et al, 2022). A British multicenter cohort study found a significantly lower QOL in patients with MASLD than in the control group. Patients with bland MASLD were as impaired in their QOL as patients with advanced fibrosis (Papatheodoridi et al, 2023). Altogether, our results support the view of HS being a broader systemic inflammatory condition.

Within the entire HS cohort, dyslipidemia was detected by laboratory chemistry in 36% of patients. Patients with HS are thus 1.5–2 times more likely to be affected than the normal population (Halcox et al, 2017; Jaross et al, 1994). Given the serious cardiovascular risks associated with long-term untreated dyslipidemia (Parhofer and Laufs, 2019) and the higher baseline risk of cardiovascular disease in HS (Tzellos et al, 2015), systematic dyslipidemia screening should be conducted for patients with HS.

In MASLD without an inflammatory component, transaminases are often within the normal range (Roeb et al, 2022), which we also observed in the present HS cohort. Of interest, 13% of patients with HS + MASLD showed elevated γ-GT levels, whereas all HS − MASLD patients were within physiological range. Duran-Vian et al (2019) found that patients with HS + MASLD had significantly higher γ-GT levels than skin-healthy control patients with MASLD. In addition, γ-GT showed the strongest correlation of all laboratory parameters with the CAP value (r = 0.578; *P* < .001). Canbay et al (2019) proved a strong association between γ-GT and MASLD. Therefore, our results emphasize the urgency of further research on whether γ-GT values in the upper normal range may already indicate MASLD in patients with HS.

A core task of this study was the search for a simple, practical algorithm for the detection of steatosis in patients with HS. Of the 3 investigated scores, the FLI has the best predictive power for MASLD in our HS cohort. Just 1 of the recent studies on MASLD prevalence among patients with HS used noninvasive liver score but employed only NFS and Fib-4 indices, which had not been investigated for accuracy (Omari et al, 2025). The sensitivity of the NFS in the present HS cohort was only 14.3%, in contrast to existing literature reporting up to 67% (Roeb et al, 2022). The most sensitive parameter for liver cell damage, γ-GT, is not used in this score. The Fib-4 index also turned out to be unsuitable for fibrosis screening in patients with HS. The sensitivity was 0%, although fibrosis was present in 15% of patients with HS + MASLD, as measured by TE. The sensitivity and specificity values described earlier (up to 86 and 95%, respectively) could not be confirmed (Kaswala et al, 2016; Nones et al, 2017), but the patients examined in those studies had pathologically elevated transaminases, insulin resistance or diabetes mellitus, and an older age (55 ± 10 years). In a HS cohort, particularly during initial screening of young patients with predominantly normal transaminases, no benefit would be expected from scoring NFS and Fib-4 indices.

In comparison, the FLI score is intended to detect MASLD without additional inflammation. In the present HS cohort, it showed very good sensitivity and specificity. For the proposed screening algorithm, a lower cutoff >20 points were chosen, which was also tested in the implementation study to achieve the highest possible sensitivity (Bedogni et al, 2006). Because steatosis can lead to high morbidity in its late stages and has a high rate of reversibility with adequate therapy (Roeb et al, 2022), a loss of specificity associated with potential overdiagnosis is preferable to the risks of underdiagnosis. Accordingly, an age-independent cutoff can be used, which further simplifies application in everyday clinical practice. The strong correlation of the FLI with the CAP score (*P* < .001) underlines the validity of the FLI as a diagnostic tool in patients with HS.

Limitations of this study were the single-center design, the comparatively small sample size, and the lack of external validation of the proposed algorithm in an independent control cohort.

In summary, in this study, we provide evidence for a high prevalence of MASLD and fibrosis among patients with HS. Moreover, we observed an above-average prevalence of T2DM in our cohort. Among the laboratory parameters, γ-GT—although it is a nonspecific marker affected by many factors, such as age or medication, and although it is not part of validated MASLD risk scores—appears to have potential as a supportive marker in predicting MASLD in patients with HS, whereas the determination of transaminases proved to be insignificant. Importantly, we could validate the FLI as an effective and simple screening tool for everyday clinical practice and have created an easy-to-implement algorithm that incorporates noninvasive clinical and laboratory markers. In view of the high risk of MASLD in HS, a systematic screening for comorbid liver disease appears advisable.

## Materials and Methods

### Case selection and data assessment

Ninety-four consecutive patients with HS who presented at the Department of Dermatology, University Hospital Würzburg between September 2020 and July 2022 were screened, after informed consent, to participate in our cross-sectional study, which had been approved by the Ethics Committee (AZ-107/20) of the University of Würzburg. Sixty-one participants met the inclusion criteria (Figure 1 provides more details). Demographic data and pre-existing conditions were recorded, and HS disease severity was assessed using the Hurley score, the dynamic modified Sartorius Score, and the International Hidradenitis Suppurativa Severity Score System (Zouboulis et al, 2024a, 2024b, 2017). Routine laboratory values were obtained, and the Dermatology Life Quality Index was recorded (Finlay and Khan, 1994). CMRFs were assessed as outlined in Table 6. Metabolic syndrome was diagnosed according to the criteria proposed by The National Cholesterol Education Program Adult Treatment Panel III (Lipsy, 2003). Individuals with a known history of malignancy, florid infections, and/or current immunosuppressive or immunomodulatory treatment were excluded from the study. Exclusion criteria also comprised other inflammatory skin diseases, identifiable increased alcohol consumption by means of the Alcohol Use Disorders Identification Test (Saunders et al, 1993), any pre-existing liver disease, regular use of potential hepatotoxic medications, anemia (hemoglobin <8.0 g/dl) or blood transfusion within the last 4 weeks, or refusal to undergo TE of the liver.

**Table 6:** Best Set of Predictors of MASLD Risk in the Multivariable Regression Analysis

Number	Set of Predictors	Age, y	R^2^	Regression Coefficient β	*P*-Value	95% CI
1	Sex	All	0.322	−13.8	.048	−27.4 to −0.2
	Metabolic risk factor			59.7	<.001	38.1–81.3
	HS			3.2	<.001	1.5–4.8
2	Sex	<45	0.446	−26.8	.017	−48.7 to −4.9
	Metabolic risk factor			61.5	<.001	34.3–88.6
	HS			4.1	<.001	1.8–6.4
3	Sex	≥45	0.142	−4.2	ns	−21.6 to 88.9
	Metabolic risk factor			51.7	.007	14.5–88.9
	HS			2.5	.036	0.2–4.8

Abbreviations: CI, confidence interval; HS, hidradenitis suppurativa; ns, not significant.

F-test was performed for assessment of the overall model; *t*-test was used to evaluate individual predictors; multicollinearity diagnostics were used to check the independence of predictors; confidence intervals were used for estimating the precision of the coefficients.

### Clinical risk stratification for MASLD

The Fib-4 index (considering age, aspartate aminotransferase, alanine aminotransferase, and platelet count), the NFS (based on age, BMI, impaired fasting glucose/diabetes, aspartate aminotransferase, alanine aminotransferase, platelet count, and albumin) and the FLI (based on height, weight, waist circumference, triglycerides, and γ-GT) were deployed to clinically assess the risk of liver fibrosis before TE (Ahmed et al, 2024; Amer et al, 2024; Crudele et al, 2024; Lee et al, 2021; Sun et al, 2016; Zambrano-Huailla et al, 2020). The Fib-4 index is calculated according to the following formula (Shah et al, 2011):

$$\text{Fib}-4\;\text{Score}=\left({\text{age}}^{*}\times \text{aspartate}\;\text{aminotransferase}\right)\;/\;(\text{platelets}\times \sqrt{\left[\text{alanine}\;\text{aminotransferase}\right])}$$

∗It should be used with caution in patients aged <35 or >65 years because the score has been shown to be less reliable in these age groups. The Fib-4 scores with corresponding fibrosis stages are presented in Table 7.

**Table 7:** Calculation and Interpretation of the Fib-4 Score

Fib-4 Score	Approximate Fibrosis Stage
<1.45	0–1
1.45–3.25	2–3
>3.25	4–6

The NFS is calculated according to the following formula (Angulo et al, 2007):

$$\text{NFS}=-1.675+\left(0.037\times \text{age}\;\left[\text{years}\right]\right)+\left(0.094\times \text{BMI}\;\left[\text{kg}/m^{2}\right]\right)+\left(1.13\times \text{impaired}\;\text{fasting}\;\text{glucose}/\text{diabetes}\;\left[\text{yes}=1,\text{no}=0\right]\right)+\left(0.99\times \text{aspartate}\;\text{aminotransferase}/\text{alanine}\;\text{aminotransferase}\;\text{ratio}\right)\;–\;\left(0.013\times \text{platelet}\;\text{count}\;\left[\times 109/l\right]\right)\;–\;\left(0.66\times \text{albumin}\;\left[g/\text{dl}\right]\right)$$

The NFS with the correlated fibrosis severities are provided in Table 8.

**Table 8:** Calculation and Interpretation of the NAFLD Score

NAFLD Score	Correlated Fibrosis Severity
≤1.455	F0–F2
−1.455 to 0.675	Indeterminant score
>0.675	F3–F4

Abbreviation: NAFLD, nonalcoholic fatty liver disease.

The Fibrosis Severity Scale assesses the severity of fibrosis: F0 = no fibrosis, F1 = mild fibrosis, F2 = moderate fibrosis, F3 = severe fibrosis, and F4 = cirrhosis.

The FLI is calculated according to the following formula (Bedogni et al, 2006):

$$\text{FLI}=e^{y}\;/\;\left(1+e^{y}\right)\times 100$$

Where y = 0.953 × ln(triglycerides [mg/dl]) + 0.139 × BMI [kg/m^2^] + 0.718 × ln(γ-GT [U/L]) + 0.053 × waist circumference (cm) – 15.745. the FLI with corresponding risks and diagnosis is provided in Table 9.

**Table 9:** Calculation and Interpretation of the Fatty Liver Index

FLI	Risk	Diagnosis
<30	Low	Fatty liver ruled out (negative likelihood ratio = 0.2)
30 to <60	Indeterminate	Fatty liver neither ruled in nor ruled out
≥60	High	Fatty liver ruled in (positive likelihood ratio = 4.3)

Abbreviation: FLI, Fatty Liver Index.

### Assessment of MASLD

TE was used to diagnose and classify MASLD (Isabela Andronescu et al, 2018; Karlas et al, 2017; Lee and Park, 2014; Nalbantoglu and Brunt, 2014; Potts et al, 2017). Clinical diagnosis of MASLD was based on an altered TE CAP and the presence of at least 1 CMRF. TE was performed by a skilled hepatologist blinded to participants’ medical history using a FibroScan Compact 530 device (Echosens) to assess CAP (as surrogate for liver steatosis) and liver stiffness measurement (marker for liver fibrosis).

The cutoffs used to grade steatosis severity for the values obtained by TE were <230 dB/m for S0, 231–270 for S1 dB/m, 271–300 dB/m for S2, and >300 dB/m for S3, and the cutoffs to grade fibrosis severity were <8.0 kPa for ≤F1, 8–12 kPa for F2–3, and >12 kPa for F4. CAP performance might be altered by the concomitant presence of T2DM or obesity, and therefore, results were adjusted if needed (Karlas et al, 2017). As an indicator of variability, the ratio of the interquartile range was estimated. Examinations with no successful measurements after at least 10 attempts or an interquartile range >3.0 were considered failures.

### Statistical analysis

Descriptive statistics were used to summarize the data. Unless explicitly mentioned, data represent means ± SD. All variables were assessed using the Kolmogorov–Smirnov test to determine whether parametric tests could be applied. Normally distributed quantitative variables were analyzed by Student’s *t*-test or 1-way ANOVA, and those non-normally distributed were assessed by Mann–Whitney *U* test. Categorical variables were compared by chi-square or Fisher’s exact test, as appropriate. A logistic univariable analysis was initially performed to determine the differences between patients with HS and controls as well as between MASLD and non-MASLD cases. Then, a stepwise multivariable logistic regression model using the extent of fatty liver disease, measured by CAP value, as dependent variable, and covariates associated with the diagnosis and severity of MASLD and HS (age, sex, CMRFs), was performed to know the best set of predictors of MASLD risk. The association between normally distributed variables was assessed using the Pearson correlation. For non-normally distributed variables, Spearman’s rank correlation coefficient was applied. The effect sizes (r) were interpreted according to Cohen's classification: r = 0.10 indicates a weak effect, r = 0.30 indicates a medium effect, and r = 0.50 indicates a strong effect. The statistical significance level was set at *P* < .05. To assess the results of our study, we used Microsoft Excel (version 16.0.17029.20140, Microsoft) and Statistical Package for the Social Sciences for Windows (version 28.0, SPSS, Chicago, IL).

## Ethics Statement

The cross-sectional study was performed in accordance with the ethical guidelines of the Declaration of Helsinki and approved by the Ethics Committee (AZ-107/20) of the University of Würzburg. A signed informed consent was obtained from all participants prior to inclusion.

## Data Availability Statement

All relevant data are provided within the manuscript. No data from the study have been previously published.

## ORCIDs

Verena G. Frings: http://orcid.org/0000-0002-8256-7961

Maxine Gläsel: http://orcid.org/0009-0007-3161-2105

Monika Rau: http://orcid.org/0000-0003-1219-4044

Andreas Geier: http://orcid.org/0000-0002-9626-5083

Janik Fleißner: http://orcid.org/0009-0000-1435-0245

Dagmar Presser: http://orcid.org/0000-0001-8535-1309

Matthias Goebeler: http://orcid.org/0000-0001-7095-9848

Andreas Kerstan: http://orcid.org/0000-0001-6483-0191

## Conflict of Interest

DP is a member of the European Hidradenitis Suppurativa Foundation). Department of Dermatology, Venereology and Allergology, University Hospital Würzburg (with active members DP and MGo) is a healthcare provider center of the European Network for Rare and Low Prevalence Complex Skin diseases (ERN Skin) and conducted clinical studies of phases II and III on hidradenitis suppurativa for Janssen, Novartis, Sanofi, and UCB (with investigators DP and MGo). The remaining authors state no conflict of interest.
